# Epidemiologic comparisons of soccer-related injuries presenting to emergency departments and reported within high school and collegiate settings

**DOI:** 10.1186/s40621-017-0116-9

**Published:** 2017-07-03

**Authors:** Zachary Y. Kerr, Lauren A. Pierpoint, Dustin W. Currie, Erin B. Wasserman, R. Dawn Comstock

**Affiliations:** 1 Department of Exercise and Sport Science, University of North Carolina, 313 Woollen Gym CB#8700, Chapel Hill, NC 27599-8700 USA; 2 Department of Epidemiology, Colorado School of Public Health, University of Colorado Anschutz, Mail Stop B119, 13001 E 17th Pl, Aurora, CO 80045 USA; 3 Datalys Center for Sports Injury Research and Prevention, 401 W Michigan St, Suite 500, Indianapolis, IN 46202 USA; 4 Department of Pediatrics, School of Medicine, University of Colorado, Mail Stop B119, 13001 E 17th Pl, Aurora, CO 80045 USA

**Keywords:** Injury surveillance, Soccer, Epidemiology, Athletic training

## Abstract

**Background:**

Few studies compare sports injury patterns in different settings. This study described the epidemiology of soccer injuries presenting to emergency departments (EDs) and compared injuries presenting to EDs to injuries presenting to collegiate and high school athletic trainers (ATs).

**Methods:**

Soccer-related injuries (product code 1267) in the National Electronic Injury Surveillance System (NEISS) that were sustained by individuals at least two years of age in 2004–2013 were included. High School Reporting Information Online (HS RIO) data for high school soccer injuries during the 2005/06–2013/14 academic years were compared to NEISS data for those aged 14–17 years in 2005–2013. National Collegiate Athletic Association Injury Surveillance Program (NCAA-ISP) data for collegiate soccer injuries during the 2009/10–2013/14 academic years were compared to NEISS data for those aged 18–22 years in 2009–2013. All datasets included weights to calculate national estimates. Injury proportion ratios (IPRs) with 95% confidence intervals (CIs) compared nationally estimated injury distributions between the HS RIO/NCAA-ISP and NEISS data subsets.

**Results:**

During the study period, 63,258 soccer-related injuries were captured by NEISS, which translates to an estimated 2,039,250 injuries seen at US EDs nationwide. Commonly injured body parts included the head/face (19.1%), ankle (17.6%), hand/wrist (15.3%), and knee (12.2%). Common diagnoses included sprains/strains (34.0%), fractures (22.2%), and contusions (17.7%). Compared to their respective age ranges in NEISS, sprains/strains comprised a larger proportion of injuries in HS RIO (48.3% vs. 33.7%; IPR = 1.38; 95% CI: 1.33, 1.42) and NCAA-ISP (51.3% vs. 37.0%; IPR = 1.39; 95% CI: 1.31, 1.46). In contrast, fractures comprised a smaller proportion of injuries in HS RIO than in NEISS (7.5% vs. 18.6%; IPR = 0.43; 95% CI: 0.39, 0.47) and NCAA-ISP (2.8% vs. 15.7%; IPR = 0.18; 95% CI: 0.14, 0.22).

**Conclusions:**

ATs more commonly reported injuries that are easily diagnosed and treated (e.g., sprains/strains); EDs more commonly reported injuries with longer recovery times and rehabilitation (e.g., fractures). Although ED surveillance data can identify the most severe sports-related injuries, high school and college sports surveillance may better describe the breadth of sports-related injuries. Our findings may provide further support for school-based sports medicine professionals, but further research is needed to comprehensively examine the potential economic and health-related benefits.

## Background

Soccer is one of the most popular sports worldwide, with an estimated 265 million participants in 2006. Although the popularity of soccer is perceived to be larger outside of the United States (US), estimates of participation in the US are high, ranging from 17.6 to 24.5 million (Kunz 2007; Sports and Fitness Industry Association 2013). Soccer is a popular high school and collegiate sport in the US. During the 2013/14 academic year, the National Federation of State High School Associations (NFHS) reported 417,419 boys and 374,564 girls played high school soccer and the National Collegiate Athletic Association (NCAA) reported 23,602 males and 26,358 females played collegiate soccer (National Federation of High Schools 2013; National Collegiate Athletic Association 1981).

Like all sports and recreational activities, although playing soccer provides several positive health benefits, it also poses a risk of injury. With such large numbers of participants, the injury risk associated with playing soccer poses a public health concern. Previously published works by US researchers evaluating population-based data on soccer-related injuries have been mostly limited to youth populations or are over a decade old (Adams & Schiff 2006; Leininger et al. 2007; Finch et al. 1998; Kelly et al. 2001; Walters et al. 2014; Smith et al. 2016). An examination of the epidemiology of soccer injuries across the lifespan using more recent data from varied clinical settings should help drive the development of age-appropriate interventions to reduce the incidence and severity of injury.

One intervention that may assist in mitigating the severity of injury is presence of a sports medicine clinician such as a physician or athletic trainer (AT). Although the presence of an onsite sports medicine professional has long been advocated, coverage at soccer activities varies by level of play (American Medical Association 1998; Fletcher et al. 2014; Kerr et al. 2014a). The NCAA Sports Medicine Handbook advocates for all member institutions to provide appropriate AT coverage across all sports and divisions (National Collegiate Athletic Association 2014). ATs are not required at the high school level and data suggest that even though 70% of high schools have access to some form of AT coverage, only 55% of student-athletes have access to a full-time AT (Pryor et al. 2015). Having ATs available onsite: allows participants to have injuries immediately diagnosed, treated, and managed; mitigates resulting injury severity; and reduces the need to present to emergency departments (EDs) or other healthcare facilities with higher associated costs (Wham et al. 2008; 2010). Thus, it should be expected that soccer injuries presenting for treatment to ATs in the collegiate or high school setting will differ from injuries presenting for treatment in the ED setting.

To our knowledge, to date there have been no examinations of soccer-related injuries presenting to different clinical settings across the high school and collegiate age span. One study compared the epidemiology of basketball-related injuries presenting to EDs to those reported by ATs in a high school setting (Fletcher et al. 2014), finding that EDs reported larger proportions of severe injuries such as fractures than high schools. However, the study did not consider additional levels of competition. This study builds on the findings of prior studies by examining the epidemiology of soccer-related injuries across the lifespan. First, we examined soccer-related injuries seen at US EDs from 2004 to 2013 to identify epidemiologic trends and to develop targeted recommendations to reduce risk of injury. Second, the ED injury surveillance data from high school- and college-aged individuals was respectively compared to injury surveillance data originating from the National High School Reporting Information Online (HS RIO) and the National Collegiate Athletic Association (NCAA) Injury Surveillance Program (ISP). The two objectives of this study were to 1) describe the epidemiology of soccer injuries across the age span presenting to EDs and 2) compare the epidemiology of soccer injuries presenting to the ED to soccer injuries presenting to collegiate and high school ATs.

## Methods

### Data sources

In this study, soccer injury data from three large national surveillance systems were evaluated. This study is exempt from human subjects review as it uses previously collected data that were de-identified.

#### National electronic injury surveillance system

ED data were obtained from the US Consumer Product Safety Commission’s (CPSC) National Electronic Injury Surveillance System (NEISS), which has been previously described in detail (US Consumer Product Safety Commission 2017). The NEISS data originates from a stratified probability sample of approximately 100 US hospitals with at least six beds and a 24-h ED. ED records that were completed by various ED staff members were reviewed daily with demographic, injury, and treatment information logged into the NEISS database by a designated NEISS coordinator (a trained individual designated by the hospital that was either an ED staff member or contracted by CPSC). Statistical weights provided by CPSC enable generation of national estimates of the number of injuries treated in all EDs.

The sample includes data from soccer-related injuries (product code 1267) sustained by individuals at least 2 years of age from January 1, 2004 to December 31, 2013 (*n* = 63,288). We excluded individuals under 2 years of age as we believed that it would not be feasible for an individual at that age to participate in soccer. Further, narratives reviews were conducted for individuals aged 2–4 years of age or over 50 years of age to exclude those clearly unassociated with playing soccer (e.g., being held by parent who was playing soccer; spectator hit by soccer ball at grandchild’s practice) resulted in 30 exclusions, leading to a final *n* = 63,258 cases.

#### HS RIO

HS RIO, which has been previously described, is an Internet-based sport-related injury surveillance system that has captured injury and exposure data from a sample of 100 nationally representative high schools since the 2005/06 academic year (Centers for Disease Control and Prevention 2006; Rechel et al. 2008). Eligible high schools had one or more ATs with a valid email address. ATs from participating high schools reported injury incidence and athlete exposure information weekly throughout each academic year using a secure website. For each injury, ATs completed a detailed injury report on the injured athlete (age, height, weight, etc.), the injury (site, diagnosis, severity, etc.), and the injury event (activity, mechanism, etc.). ATs were able to view and update previously submitted reports as needed with new information (e.g., time loss).

A reportable injury in HS RIO was defined as an injury that: (1) occurred as a result of participation in an organized practice or competition; (2) required medical attention by an AT or physician; and (3) resulted in restriction of the student-athlete’s participation from soccer for one or more days beyond the day of injury. In addition, beginning in 2007/08, ATs were asked to include any concussion, fracture, or dental injury occurring in an organized practice or game, regardless of time loss. Only the principal injury was captured. National injury estimates are calculated from HS RIO injury count data using a weighting algorithm based on the inverse probability of participant schools’ selection into the study (based on geographic location and school size).

#### NCAA-ISP

The NCAA-ISP, which has been previously described in detail, is an Internet-based surveillance system that depends on a convenience sample of NCAA institutions with ATs voluntarily reporting injury and exposure data (Kerr et al. 2014b). Participation in the NCAA-ISP, while voluntary, is available to all NCAA institutions. For each injury event, ATs completed a detailed event report on the injury or condition (e.g., site, diagnosis) and the circumstances [e.g., activity, mechanism, event type (i.e., competition or practice)]. ATs were able to view and update previously submitted information as needed during the course of a season. In addition, ATs also provided the number of student-athletes participating in each practice and competition. The data were stripped of any identifiers and personally identifiable information (PII) retaining only relevant variables.

During the 2009/10–2013/14 academic years, the Datalys Center introduced new components to the web-based surveillance system to improve process flow; a common data element (CDE) standard was implemented (Kerr et al. 2014b). The CDE standard allows data to be gathered from various EMR/injury documentation applications. The CDE export standard allowed ATs to document injuries as they normally would as part of their daily clinical practice, as opposed to asking them to report injuries solely for purposes of participation in an injury surveillance program. Then, the de-identified and HIPAA-compliant data were sent to the Datalys Center where it was examined by data quality control staff and a verification engine (VE) (Kerr et al. 2014b).

A reportable injury in the NCAA-ISP was defined as an injury that: (1) occurred as a result of participation in an organized practice or competition; and (2) required medical attention by a certified athletic trainer (AT) or physician. The NCAA-ISP included all injuries regardless of time loss, and multiple injuries that occurred from one injury event. To calculate national estimates of the number of injuries, post-stratification sample weights, based upon sport, division, and academic year, were applied to each reported injury and athlete-exposure. Post-stratification sample weights were calculated using the formula:

$$ weigh{t}_{ijk}={\left(\frac{Number\kern0.5em  of\kern0.5em  ISS\kern0.5em  School{s}_{ijk}}{Number\kern0.5em  of\kern0.5em  Sponsoring\kern0.5em  School{s}_{ijk}}\right)}^{-1} $$

where weight^ijk^ is the weight for the i^th^ sport of the j^th^ division in the k^th^ year. Weights were further adjusted to correct for underreporting, according to findings from Kucera et al. (Kucera et al. 2011), which estimated that the ISP captured 88.3% of all time-loss medical-care injury events. Weighted counts were scaled up by a factor of (0.883)^-1^.

### Study variables

Variables were coded so that all three surveillance systems presented injury data in similar manners. Body parts injured were categorized as: Head/face, Neck, Shoulder, Arm/elbow, Hand/wrist, Trunk, Thigh/upper leg, Knee, Lower leg, Ankle, Foot, and Other. Diagnoses were categorized as: Concussion, Contusion, Dislocation, Fracture, Laceration, Sprain/strain, and Other. The NEISS data was also categorized by disposition: Released, Hospitalized, Fatality, and Other (including transferred, left without being seen, not recorded, etc.). Age groups (in years) for NEISS data were categorized as: 2–4, 5–10, 11–13, 14–17, 18–22, 23–29, 30–39, 40–49, and 50 + .

### Statistical analyses

Data were analyzed with SAS (version 9.3; SAS Institute, Cary, NC). For NEISS data, we computed injury rates overall and by age and sex. Denominator data originated from intercensal population estimates provided by the US Census Bureau (US Census Bureau. Population and Housing Unit Estimates 2017). Injury rates were presented per 1000 US Census intercensal population estimate. Injury rates ratios (IRRs) compared rates by sex and age group. Trends for injury rates over time were analyzed using linear regression. Injury proportion ratios (IPRs) with 95% confidence intervals (CIs) were calculated to compare injury proportions between age groups. The following is an example of an IPR comparing the proportion of ankle injuries reported in females versus males within the NEISS data.

$$ \mathrm{I}\mathrm{P}\mathrm{R}=\frac{\left(\frac{{\displaystyle \sum}\mathrm{ankle}\ \mathrm{in}\mathrm{juries}\ \mathrm{reported}\ \mathrm{in}\ \mathrm{females}\ \mathrm{in}\ \mathrm{NEISS}\ \mathrm{data}}{{\displaystyle \sum}\mathrm{total}\ \mathrm{in}\mathrm{juries}\ \mathrm{reported}\ \mathrm{in}\ \mathrm{females}\ \mathrm{in}\ \mathrm{NEISS}\ \mathrm{data}}\right)}{\left(\frac{{\displaystyle \sum}\mathrm{ankle}\ \mathrm{in}\mathrm{juries}\ \mathrm{reported}\ \mathrm{in}\ \mathrm{males}\ \mathrm{in}\ \mathrm{NEISS}\ \mathrm{data}}{{\displaystyle \sum}\mathrm{total}\ \mathrm{in}\mathrm{juries}\ \mathrm{reported}\ \mathrm{in}\ \mathrm{males}\ \mathrm{in}\ \mathrm{NEISS}\ \mathrm{data}}\right)} $$

To compare NEISS data to HS RIO and NCAA-ISP data, we restricted NEISS injury data to those cases aged 14–17 and 18–22 years, respectively. IPRs compared the distributions of body parts injured and diagnoses between the 14 and 17 year old NEISS cases and the HS RIO cases, and between the 18 and 22 year old NEISS cases and the NCAA-ISP cases. All 95% CIs not containing 1.00 and Linear trend *P*-values <0.05 were considered statistically significant.

## Results

### Injury incidence among those presenting to US EDs

During the study period, 63,258 soccer-related injuries were captured by NEISS, which translates to an estimated 2,039,250 injuries seen at US EDs nationwide (Table 1), and an overall injury rate of 0.69/1000 population. The mean age of those injured was 17 ± 9 years (range: 2–95 years). An estimated 61.3% of US soccer-related injuries occurred in males. The injury rate in males (0.86/1000 population) was 1.64 times that of females (0.52/1000 population; 95%CI: 1.61, 1.67). Most injuries were sustained by individuals aged 14–17 years (33.7%), 10–13 years (20.7%), 5–9 years (13.0%), and 18–22 years (11.5%). However, the injury rate was higher among those aged 14–17 years than all other ages (4.01 vs. 0.48/1000 population; IRR = 8.31; 95%CI: 8.17, 8.45).

**Table 1:** Soccer-related injuries treated in US emergency departments from 2004 to 2013^a^

	Actual counts		National estimates^b^	
Variable	n	%	n	%
Sex				
Male	39,623	62.6	1,249,650	61.3
Female	23,629	37.4	789,447	38.7
Unknown	6	<0.1	153	<0.1
Body Part				
Head/face	12,495	19.8	389,098	19.1
Neck	742	1.2	22,636	1.1
Shoulder	2,983	4.7	95,826	4.7
Arm/elbow	4,792	7.6	130,865	6.4
Hand/wrist	9,483	15.0	312,371	15.3
Trunk	4,367	6.9	140,000	6.9
Thigh/upper leg	707	1.1	23,197	1.1
Knee	7,528	11.9	249,572	12.2
Lower leg	3,703	5.9	115,154	5.7
Ankle	10,633	16.8	359,583	17.6
Foot	5,387	8.5	187,046	9.2
Other^c^	438	0.7	13,904	0.7
Diagnosis				
Concussion	2,981	4.7	84,606	4.2
Contusion	10,673	16.9	361,302	17.7
Dislocation	1,740	2.8	58,756	2.9
Fracture	14,750	23.3	452,411	22.2
Laceration	3,571	5.7	120,190	5.9
Sprain/strain	20,604	32.6	693,211	34.0
Other^d^	8,939	14.1	268,775	13.2
Disposition				
Released	61,313	96.9	1,989,373	97.6
Hospitalized	1,427	2.3	31,325	1.5
Fatality	6	<0.1	151	<0.1
Other^e^	512	0.8	18,401	0.9
Total	63,258	100.0	2,039,250	100.0

^a^Does not include cases under 2 years of age

^b^National estimates were calculated by applying statistical weights provided by the US Consumer Product Safety. Commission’s National Electronic Injury Surveillance System to actual case counts

^c^Includes internal injuries, injuries not recorded, etc

^d^Includes burns, ingesting foreign objects, unrecorded diagnoses, etc

^e^Includes transferred, left without being seen, not recorded, etc

**Fig. 1:**
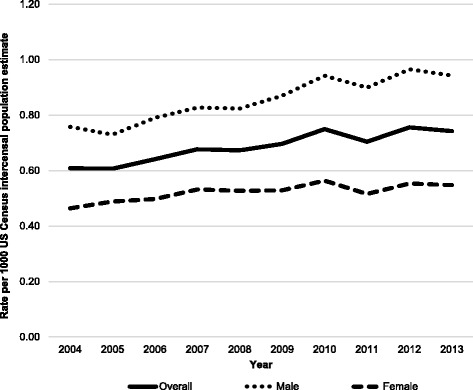
Rates of soccer-related injuries treated in US emergency departments from 2004 to 2013 by sex and year^a^. ^a^National estimates were calculated by applying statistical weights provided by the US Consumer Product Safety Commission’s National Electronic Injury Surveillance System to actual case counts. Linear trend *P*-values: Overall *P*< 0.001; Male *P*<0.001; Female *P*=0.003

**Fig. 2:**
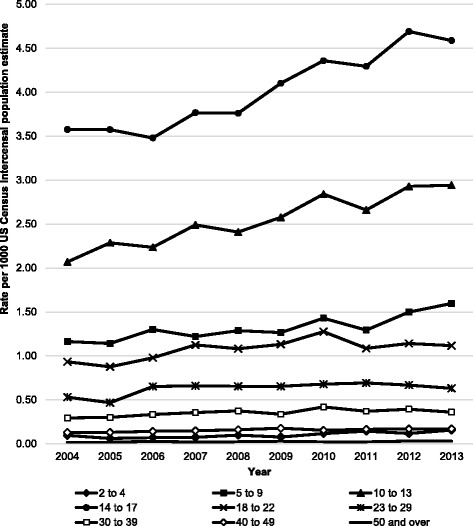
Rates of soccer-related injuries treated in US emergency departments from 2004 to 2013 by age group and year^a^. ^a^ National estimates were calculated by applying statistical weights provided by the US Consumer Product Safety Commission’s National Electronic Injury Surveillance System to actual case counts. Linear trend *P*-values: 2 to 4 *P*< 0.001; 5 to 9 *P*< 0.001; 10 to 13 *P*< 0.001; 14 to 17 *P*< 0.001; 18 to 22 *P*=0.001; 23 to 29 *P*=0.006; 30 to 39 *P*< 0.001; 40 to 49 *P*< 0.001; 50 and over *P*=0.01

The number of estimated injuries ranged from a low of 173,313 in 2004 to a high of 228,776 in 2013. However, the smallest and largest yearly injury rates were in 2005 (0.61/1000 population) and 2012 (0.76/1000 population), respectively. There was a 22.0% increase in the overall injury rate (Linear trend *P* < 0.001), as well as 24.5% and 18.0% increases in male and female injury rates, respectively (Linear trend *P* < 0.001 and *P* = 0.003, respectively) (Fig. 1). All age groups exhibited linear trends that indicated increasing injury rates (Fig. 2). The largest relative increases were seen in individuals aged 50+ years (66.5%) and 2–4 years (62.1%), although they both were the lowest overall rates of all age groups; the largest absolute increases were seen in individuals aged 14–17 years (1.01) and 10–13 years (0.87). Within age groups, the largest male versus female rate ratios between sexes were seen among individuals aged 23–29 years (1.04 vs. 0.21/1000 population; IRR = 4.95; 95% CI: 4.61, 5.32), 30–39 years (0.57 vs. 0.13/1000 population; IRR = 4.29; 95% CI: 3.96, 4.65), and 40–49 years (0.25 vs. 0.06/1000 population; IRR = 4.59; 95% CI: 4.06, 5.19).

### Injury characteristics among those presenting to US EDs

The most common body parts injured were the head/face (19.1%), ankle (17.6%), hand/wrist (15.3%), and knee (12.2%). Females had a larger proportion of ankle injuries (20.5%; IPR = 1.30; 95% CI: 1.25, 1.35) than males (15.8%). The most common diagnoses were sprains/strains (34.0%), fractures (22.2%), and contusions (17.7%). Compared to females, males had higher proportions of fractures (24.6% vs. 18.3%; IPR = 1.34; 95% CI: 1.27, 1.42) and lacerations (8.0% vs. 2.5%; IPR = 3.17; 95% CI: 2.81, 3.57). Compared to males, females had a higher proportion of concussions (5.2% vs. 3.5%; IPR = 1.48; 95% CI: 1.26, 1.74).

The majority of injuries presenting to the ED were treated and released (97.6%) (Table 1). An estimated 151 soccer-related deaths occurred nationwide, based on six fatalities captured by NEISS during the study period. Five deaths occurred in males; three deaths were due to cardiac arrest while playing soccer.

### Age differences among those presenting to US EDs

Body part injured and injury diagnosis differed by age (Table 2). Compared to all other age groups, individuals aged 2–4 years had a higher proportion of head/face (30.9% vs. 19.0%; IPR = 1.63; 95% CI: 1.33, 1.99) and arm/elbow injuries (19.9% vs. 6.3%; IPR = 3.14; 95% CI: 2.62, 3.77). Compared to all other age groups, individuals aged 14–22 years had a higher proportion of concussions (5.8% vs. 2.7%; IPR = 2.13; 95% CI: 1.90, 2.38).

**Table 2:** National estimates of soccer-related injuries treated in US emergency departments from 2004 to 2013 by age group^a^

	Age, y								
	2 to 4	5 to 9	10 to 13	14 to 17	18 to 22	23 to 29	30 to 39	40 to 49	50 and over
	(*n* = 12,146)	(*n* = 264,307)	(*n* = 422,637)	(*n* = 688,057)	(*n* = 234,997)	(*n* = 184,017)	(*n* = 142,598)	(*n* = 67,511)	(*n* = 22,981)
Variable	%	%	%	%	%	%	%	%	%
Body Part									
Head/face	30.9	17.2	14.0	22.6	22.6	18.5	16.9	14.9	16.4
Neck	2.2	1.3	1.2	1.2	1.0	0.7	0.9	1.0	1.2
Shoulder	5.7	3.4	4.1	4.2	5.3	6.2	6.3	7.9	6.6
Arm/elbow	19.9	13.5	9.5	4.1	2.6	4.5	4.4	3.4	6.2
Hand/wrist	10.3	24.3	23.3	12.7	9.1	8.5	8.8	11.9	14.5
Trunk	3.9	5.9	5.5	7.1	6.3	8.1	8.6	10.9	11.9
Thigh/upper leg	2.2	1.1	0.9	1.1	1.1	1.3	1.6	1.7	1.8
Knee	5.1	7.6	10.1	12.8	14.6	17.4	14.1	13.7	11.2
Lower leg	5.9	4.2	4.4	5.5	6.1	5.9	8.8	10.7	7.7
Ankle	6.1	10.9	16.6	19.6	21.2	19.8	18.5	13.7	12.6
Foot	6.4	9.8	10.0	8.3	9.1	8.8	10.6	9.8	8.5
Other^b^	1.2	0.8	0.5	0.7	1.0	0.5	0.5	0.4	1.3
Total	100.0	100.0	100.0	100.0	100.0	100.0	100.0	100.0	100.0
Diagnosis									
Concussion	2.6	2.5	3.6	6.4	4.1	2.1	1.8	2.4	2.4
Contusion	18.7	19.9	19.2	19.1	15.5	13.8	13.8	13.7	13.3
Dislocation	1.3	0.9	1.2	2.7	4.3	5.7	5.3	4.7	3.6
Fracture	31.3	29.7	28.2	17.9	16.2	20.2	21.6	23.0	26.7
Laceration	11.7	5.5	3.5	4.9	9.1	8.8	8.7	6.6	7.2
Sprain/strain	14.1	26.6	32.3	35.1	37.7	37.7	37.5	36.8	30.5
Other^c^	20.2	14.9	12.0	13.9	13.1	11.7	11.3	12.8	16.4
Total	100.0	100.0	100.0	100.0	100.0	100.0	100.0	100.0	100.0

^a^National estimates were calculated by applying statistical weights provided by the US Consumer Product Safety Commission’s National Electronic Injury Surveillance System to actual case counts

^b^Includes internal injuries, injuries not recorded, etc

^c^Includes burns, ingesting foreign objects, unrecorded diagnoses, etc

### Comparisons with HS RIO and NCAA-ISP data

**Fig. 3:**
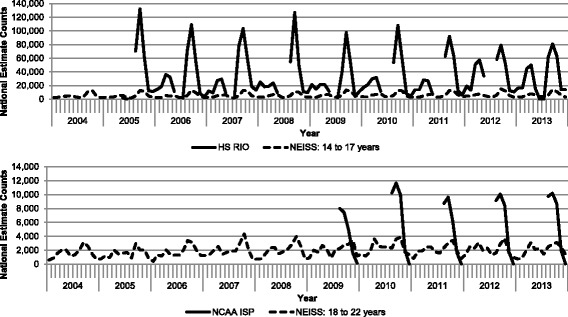
National estimate of soccer-related injury incidence for National Electronic Injury Surveillance System (NEISS), High School Reporting Information Online (HS RIO), and National Collegiate Athletic Association Injury Surveillance Program (NCAA-ISP), by month, 2004–2013^a^. NOTE: Y-Axis for both scales differ. Data collection for HS RIO and NCAA-ISP occurred only during the school-sanctioned soccer seasons, so the lines are not continuous. ^a^ National estimates were calculated by applying statistical weights provided by the US Consumer Products Safety Commisions’s National Electronic Injury Surveillance System to actual case counts

Soccer-related injuries presenting to EDs had seasonal peaks similar to peaks observed in HS RIO and the NCAA-ISP (Fig. 3). However, the nationally estimated incidence of injuries presenting to high school and collegiate ATs based on the HS RIO and the NCAA-ISP databases were generally higher than the nationally estimated incidence of injuries presenting to EDs based on the NEISS dataset. Additionally, the national estimates for injuries for high school student-athletes was larger than that for NCAA student-athletes.

Distributions of body parts injured and diagnoses differed between those presenting to EDs vs. school ATs (i.e., NEISS: 14–17 year old vs. HS RIO; NEISS: 18–22 years old vs. NCAA-ISP; Table 3). For example, compared to their respective age ranges in NEISS, thigh/upper leg injuries comprised a larger proportion of injuries in HS RIO (15.3% vs 1.1%; IPR = 14.06; 95% CI: 12.13, 16.30) and NCAA-ISP (27.0% vs. 1.2%; IPR = 21.85; 95% CI: 16.40, 29.10). Compared to their respective age ranges in NEISS, concussions comprised a larger proportion of injuries in HS RIO (17.9% vs. 7.6%; IPR = 2.75; 95% CI: 2.55, 2.96) and NCAA-ISP (5.9% vs. 4.6%; IPR = 1.30; 95% CI: 1.05, 1.59); sprains/strains also comprised a larger proportion of injuries in HS RIO (48.3% vs. 33.7%; IPR = 1.38; 95% CI: 1.33, 1.42) and NCAA-ISP (51.3% vs. 37.0%; IPR = 1.39; 95% CI: 1.31, 1.46). In contrast, fractures comprised a smaller proportion of injuries in HS RIO (7.5% vs. 18.6%; IPR = 0.43; 95% CI: 0.39, 0.47) and NCAA-ISP (2.8% vs. 15.7%; IPR = 0.18; 95% CI: 0.14, 0.22).

**Table 3:** Proportions of body sites and diagnoses of soccer-related injuries treated in US emergency departments from 2004 to 2013 (NEISS) and seen by athletic trainers in high school (HS RIO) and collegiate (NCAA-ISP) settings^a^

	14–17 year olds (2005–2013)			18–22 year olds (2009–2013)		
	HS RIO,	NEISS,		NCAA-ISP,	NEISS,	
Variable	n (%)	n (%)	IPR (95%CI)^b^	n (%)	n (%)	IPR (95%CI)^c^
Body Part						
Head/face	706571 (20.9)	4647 (24.2)	0.92 (0.87, 0.97)*	15379 (10.8)	30147 (23.5)	0.46 (0.41, 0.52)*
Neck	17584 (0.5)	256 (1.3)	0.45 (0.32, 0.63)*	615 (0.4)	1196 (0.9)	0.46 (0.25, 0.84)*
Shoulder	71693 (2.1)	857 (4.5)	0.50 (0.42, 0.59)*	3916 (2.7)	6760 (5.3)	0.52 (0.41, 0.67)*
Arm/elbow	44904 (1.3)	937 (4.9)	0.32 (0.26, 0.40)*	1164 (0.8)	3185 (2.5)	0.33 (0.22, 0.50)*
Hand/wrist	143173 (4.2)	2281 (11.9)	0.34 (0.30, 0.39)*	4859 (3.4)	12158 (9.5)	0.36 (0.29, 0.44)*
Trunk	124860 (3.7)	1407 (7.3)	0.52 (0.45, 0.59)*	7598 (5.3)	8512 (6.6)	0.80 (0.66, 0.97)*
Thigh/upper leg	517233 (15.3)	207 (1.1)	14.06 (12.13, 16.30)*	38543 (27.0)	1589 (1.2)	21.85 (16.40, 29.10)*
Knee	557899 (16.5)	2367 (12.3)	1.29 (1.20, 1.38)*	22062 (15.5)	18580 (14.5)	1.07 (0.96, 1.20)
Lower leg	248938 (7.4)	1132 (5.9)	1.34 (1.21, 1.49)*	12699 (8.9)	7526 (5.9)	1.52 (1.28, 1.80)*
Ankle	693323 (20.6)	3592 (18.7)	1.05 (0.99, 1.11)	22740 (15.9)	26909 (20.9)	0.76 (0.69, 0.84)*
Foot	216553 (6.4)	1422 (7.4)	0.79 (0.71, 0.88)*	10471 (7.3)	10381 (8.1)	0.91 (0.77, 1.07)
Other^d^	31089 (0.9)	137 (0.7)	1.26 (0.90, 1.78)	2697 (1.9)	1579 (1.2)	1.54 (1.00, 2.35)*
Diagnosis						
Concussion	604371 (17.9)	1459 (7.6)	2.75 (2.55, 2.96)*	8445 (5.9)	5865 (4.6)	1.30 (1.05, 1.59)*
Contusion	413733 (12.3)	3276 (17.0)	0.67 (0.62, 0.72)*	23041 (16.1)	17841 (13.9)	1.16 (1.04, 1.31)*
Dislocation	44768 (1.3)	492 (2.6)	0.49 (0.39, 0.62)*	776 (0.5)	5540 (4.3)	0.13 (0.08, 0.20)*
Fracture	254397 (7.5)	3575 (18.6)	0.43 (0.39, 0.47)*	3996 (2.8)	20183 (15.7)	0.18 (0.14, 0.22)*
Laceration	36898 (1.1)	917 (4.8)	0.22 (0.18, 0.28)*	1988 (1.4)	10592 (8.2)	0.17 (0.12, 0.23)*
Sprain/strain	1628374 (48.3)	6494 (33.7)	1.38 (1.33, 1.42)*	73262 (51.3)	47595 (37.0)	1.39 (1.31, 1.46)*
Other^e^	392282 (11.6)	3029 (15.7)	0.78 (0.72, 0.85)*	31234 (21.9)	20906 (16.3)	1.35 (1.22, 1.48)*

^a^National estimates were calculated by applying statistical weights provided by the US Consumer Product Safety Commission’s National Electronic Injury Surveillance System to actual case counts

^b^Compares HS RIO to NEISS; * indicates 95%CI does not contain 1.00

^c^Compares NCAA-ISP to NEISS; * indicates 95%CI does not contain 1.00

^d^Includes internal injuries, injuries not recorded, etc

^e^Includes burns, ingesting foreign objects, unrecorded diagnoses, etc

## Discussion

Utilizing data from three large national surveillance systems, this was the first study to 1) describe the epidemiology of soccer injuries across the age span presenting to EDs and 2) compare the epidemiology of injuries presenting to the ED to injuries presenting to collegiate and high school ATs. Previous studies by US researchers examining soccer-related injuries that utilized population-based data were largely restricted to analyses of youth (Adams & Schiff 2006; Leininger et al. 2007; Walters et al. 2014), were not specific to soccer (Finch et al. 1998; Kelly et al. 2001), and were approximately a decade old (Adams & Schiff 2006; Leininger et al. 2007; Finch et al. 1998; Kelly et al. 2001). Our study used national estimates to analyze soccer-related injuries in individuals aged 2 years and older that presented in US EDs during 2004–2013, and compared those patterns with two widely used sport surveillance systems, HS RIO and NCAA-ISP. We found that the patterns of soccer injuries not only differed across the age span, but also differed by clinical setting. Thus, our findings contribute important information that could be used to drive the development of targeted, setting-specific injury prevention efforts.

### Epidemiology of soccer injuries across the Age span presenting to EDs

Our findings paralleled those seen in previous research (Adams & Schiff 2006; Leininger et al. 2007; Finch et al. 1998). A large proportion of soccer injuries presenting to EDs were to the lower extremity, although numerous wrist/hand injuries also presented. Common diagnoses included sprains/strains, fractures, and contusions. Rates and patterns of injury differed by age and sex, with males having a higher rate of injury than females overall and the 14–17 year old age group having the highest rates of injuries compared to all other age groups. The 14–17 year old age group also had the largest absolute injury rate increase during the study period. This age group may have the highest rates of injury because participation opportunities are abundant, with 791,983 participating in high school soccer during the 2013/14 academic year alone (National Federation of High Schools 2013), continued increases in participation across time (National Federation of High Schools 2013), and levels of play, quality of coaching, and safety of playing facilities vary widely across high school, recreational, travel, etc. leagues. However, we acknowledge the limitations of utilizing a rate denominator based on intercensal estimates (US Census Bureau. Population and Housing Unit Estimates 2017), as these undoubtedly include individuals that do not participate in soccer activities. Availability of annual age- and sex-specific sports participation data are limited. Improving the quality and availability of such participation data would greatly benefit future studies of population injury risk. Thus, youth sports governing bodies should be encouraged to track and make publicly available accurate participation data. Such data are also important given the recent ban on heading in youth players ≤10 years old by the United States Soccer Federation (US Soccer 2015).

### Sex differences

Previous literature utilizing HS RIO and NCAA-ISP data suggest that in the school sports setting in many gender-comparable sports, females consistently had higher injury rates than males, overall and within certain injury types (e.g., concussions, ACL tears) (Hootman et al. 2007; Comstock et al. 2013). However, the ED data evaluated in this study showed males consistently had higher injury rates than females, overall and by age group. This could reflect differences in the respective populations presenting to different clinical settings in regards to true injury incidence or reporting. For example, males outside of the high school setting may sustain soccer-related injuries at a higher rate than females. Or, males may sustain more severe injuries than females and therefore may more frequently present to emergency departments (Fletcher et al. 2014). However, research has also noted that cultural differences may be associated with injury reporting (Gessel et al. 2007). Females may be more willing to disclose injuries and thus get immediate treatment; males on the other hand may only report injuries when they are more severe and require ED intervention. Our findings emphasize the need to continue examining the biomechanical and behavioral effects that may be associated with injury incidence and reporting across all ages. Nonetheless, our findings may also be associated with variations existing among data collection. For example, HS RIO and NCAA-ISP only look at injury rates among actual participants in school-sanctioned practices and competitions, whereas our rates with NEISS data consider injury rates per population. Actual participation in soccer may vary by sex, which may have biased NEISS-based injury rates.

### Comparing the epidemiology of injuries presenting to the ED to injuries presenting to collegiate and high school ATs

Utilizing an ED data source such as NEISS provides opportunities to understand patterns of the most severe injuries sustained by individuals participating in sports. However, many sports-related injuries present to and are treated by sports medicine physicians and ATs when they are present at sports practices and competitions thus reducing the number of individuals presenting to EDs. New opportunities now exist to understand the entire spectrum of sports-related injuries, rather than just the most severe injuries that present to traditionally clinical settings, because the presence of sports injury surveillance systems has increased, with focused efforts seen in numerous settings, including youth leagues (Dompier et al. 2015a), high school athletics (Centers for Disease Control and Prevention 2006; Rechel et al. 2008; Dompier et al. 2015b), college athletics (Kerr et al. 2014b; Swedler et al. 2015), and within the military (Loringer et al. 2008). However, the ED may provide the easiest access to injury data in settings lacking the presence of an AT or team sports physician. Although methods of injury reporting vary, ranging from onsite ATs (Centers for Disease Control and Prevention 2006; Rechel et al. 2008; Kerr et al. 2014b; Dompier et al. 2015a; b) to the participants themselves (Swedler et al. 2015; Loringer et al. 2008), a comprehensive evaluation of data from such surveillance systems has the potential to better describe the epidemiology of sports-related injury.

In this study, we used surveillance system data gathered from EDs (NEISS) and ATs employed at high schools (HS RIO) and colleges (NCAA-ISP) to more fully examine patterns of soccer-related injuries. As seen in previous research with basketball (Fletcher et al. 2014), injuries that were more likely to be easily diagnosed and treated such as sprains/strains comprised a larger proportion of injuries reported by ATs in the high school and college settings. Meanwhile, injuries with more complex diagnosis, treatment, and rehabilitation schedules, such as fractures, comprised a larger proportion of injuries presenting to EDs. However, sprains/strains comprised the largest proportion of injuries in all three settings. Findings slightly differed when NEISS data were compared to HS RIO and NCAA-ISP; this may be due to varying injury definitions used by both surveillance systems. Because the NCAA-ISP also included injuries resulting in <24 h of time loss, it is possible that a larger proportion of injuries that were less severe were in the dataset. This would explain the larger proportion of contusions and the smaller proportion of fractures in the NCAA-ISP dataset. Thus, as injury surveillance data are used, it is important to understand how the inclusion/exclusion of certain injuries, based upon the definition used, may affect injury distributions.

As noted by Fletcher et al. (2014), EDs may be tasked with dealing with non-urgent injuries, particularly when no sports medicine clinician is available (e.g., those high schools without AT coverage) (Pitts et al. 2008) and/or when those in attendance (e.g., coaches, referees, parents) have little/no experience in injury management/treatment. Overcrowding may burden hospital resources (Trzeciak & Rivers 2003) and can be associated with higher patient mortality (Richardson 2006). Because they triage injuries and care for more minor injuries not requiring ED care, the presence of ATs mitigates the need for some ED services among student-athletes within high school and college settings. However, it is estimated that 30% of high schools lack access to ATs (Pryor et al. 2015), and as a result, patterns of reported injuries may differ between schools with and without ATs meaning many sports-related injuries that could be managed by school ATs likely still present to EDs across the US. Furthermore, although it is advocated at the NCAA level that all member institutions provide appropriate AT coverage across all sports and divisions (National Collegiate Athletic Association 2014), variations have been found in the provision of aspects of sports injury management, such as concussion baseline testing protocols (Kerr et al. 2015). It is possible that such variations based upon sport and division extend beyond this and would thus affect the care provided to athletes by medical staff. Previous findings have suggested the benefits of having sports medicine professionals onsite (Fletcher et al. 2014; Kerr et al. 2014a; Wham et al. 2008; 2010) and are coupled with support from organizations such as the American Medical Association (1998). Our findings may provide further support for school-based sports medicine professionals. However, because our study did not examine additional metrics such as financial costs, medical outcomes, etc., future research is warranted to determine the potential benefits of on-site sports medicine professionals comprehensively.

### Concussions

Females presented to EDs with a larger proportion of concussions than males. This finding follows previous research which has found higher rates and proportions of concussions among female soccer players than male soccer players (Gessel et al. 2007; Marar et al. 2012; Delaney et al. 2002; Dick et al. 2007; Agel et al. 2007). Similarly in previous HS RIO and NCAA-ISP research, the reported concussion rate in female soccer players was significantly higher than that of male soccer players (Rosenthal et al. 2014; Zuckerman et al. 2015). Reasons for the sex differences have been discussed in the literature, ranging from different styles of play (Gessel et al. 2007) to biomechanical differences (Barnes et al. 1998; Mansell et al. 2005), particularly that compared to males, females may have smaller head-to-ball ratios, weaker necks, or less total mass in their heads and necks. Previously (Collins et al. 2014), a study with high school students found that increased neck strength was associated with a decreased odds of concussion. Mechanisms of injury may also differ by sex, as high school data suggests that compared to boys, concussions among girls were more likely to occur due to contact with the playing surface or ball while boys are more commonly injured due to contact with another player (Marar et al. 2012). Continued examination of these risk factors should help drive efforts to reduce the concussion disparity between sexes while reducing overall incidence.

Concussions comprised a smaller proportion of injuries in NEISS compared to HS RIO and NCAA-ISP. It is possible that the larger proportions of concussions in the high school and collegiate settings observed here represent increased awareness via coaching and player education. Because ATs are on-site, they may be able to provide immediate management and provide concussed athletes with resources and referrals to assist in proper return-to-play (and return-to-learning). This may reduce the presence of concussions presenting to EDs. However, future research is warranted to further demonstrate the benefits of ATs, particularly with concussion management.

In recent years, concussion rates have increased, suggesting that the proportion of concussions being reported is increasing as well (Rosenthal et al. 2014). Although concussions go unreported at the high school and college settings (Kerr et al. 2014c), we are unaware of the proportion of concussions outside of these settings that go unreported. The difference in proportions of injuries that are concussions may be associated with various settings for immediate concussion management. Further, this difference may underscore the importance of having sports medicine clinicians present at practices and competitions, particularly to help identify symptomatology and remove suspected concussed athletes from participation.

### Limitations

Accurate national soccer participation data encompassing all levels of play is difficult to obtain. Furthermore, data that does exist rarely includes participation in unorganized events such as pick-up games. As a result, we utilized intercensal population estimates from the census which may have resulted in either an underestimate or overestimate of the true rates of soccer-related injuries. The generalizability of our findings may be limited. Whereas NEISS captures all soccer-related injuries from all levels of play, HS RIO and NCAA-ISP only capture events occurring during school-sanctioned practices and competitions. Variations in medical presence at the high school and college sport settings may have led to underreporting, although the NCAA Sports Medicine Handbook advocates AT coverage for all sports at its member institutions (National Collegiate Athletic Association 2014), and approximately 70% of US high school athletic programs receive some level of AT coverage (Pryor et al. 2015).

NEISS data included only those injuries that presented to EDs and may have excluded those that were or perceived to be less severe in nature. The likelihood of presenting to an ED may also be dependent on various demographic- and injury-related factors. The NEISS cases were selected on the basis of case narratives, some of which are not detailed and thus we may have included some cases that did not actually occur during active soccer participation; case-by-case examinations were only performed at the extreme age ranges. The NEISS cases also lacked follow-up detail provided in HS RIO or NCAA-ISP, such as injury mechanism, symptomology, and severity. Additionally, it is unknown if NEISS cases received treatment from an AT prior to presenting to the ED. Although all three surveillance systems relied upon the expertise of medical staff, all of whom were trained for data collection, it is still possible that there were variations in the reporting and diagnosis of injuries.

Last, it is important to highlight that our analyses computed effect estimates that have been discussed by sports injury epidemiologists in the context of their practicality and usefulness (Knowles et al. 2006; 2010; Comstock & Fields 2010). The IRR and the IPR provide different types of comparisons. Whereas the IRR answers which of the two groups being compared had a higher incidence of injury per unit of exposure, the IPR answers in which of the two groups being compared does a specific injury comprise a greater percentage of all injuries (Comstock & Fields 2010). As previously noted, the use of intercensal population estimates may have resulted in either an underestimate or overestimate of the true rates of soccer-related injuries and may create invalid comparisons to HS RIO and NCAA-ISP data. Thus, when comparing NEISS data to those of HS RIO and NCAA-ISP, we opted to use the IPR. This metric has an important role in sports injury epidemiology and these comparisons still contribute to the body of literature examining soccer-related injuries. However, it is important to understand the difference between IRRs and IPRs, and as with any statistical analysis, be cautious with interpretation.

## Conclusions

Patterns of soccer-related injuries presenting to EDs varied by age and sex, with males and 14–17 year olds having the highest injury rates. In addition, patterns of soccer-related injuries presenting to EDs and ATs in high school and collegiate settings varied. Our findings may highlight the importance of having an AT present at high school and collegiate soccer competitions and practices. Their presence can help capture injuries that otherwise might have gone undiagnosed (i.e., concussions), and provide treatment in the school setting, therefore preventing unnecessary and costly visits to the ED. However, further research is needed to comprehensively examine the potential economic- and health-related benefits of having on-site sports medicine professionals.

## Abbreviations

AT: Athletic trainer
CDE: Common data element
CI: Confidence interval
ED: Emergency department
HS RIO: High School Reporting Information Online
IPR: Injury proportion ratio
IRR: Injury rate ratio
NCAA: National Collegiate Athletic Association
NCAA-ISP: National Collegiate Athletic Association Injury Surveillance Program
NEISS: National Electronic Injury Surveillance System
NFHS: National Federation of State High School Associations
US: United States
